# Unusual Genetic Diversity Within *Thereuopoda clunifera* (Wood, 1862) (Chilopoda: Scutigeromorpha) Revealed by Phylogeny and Divergence Times Using Mitochondrial Genomes

**DOI:** 10.3390/insects16050486

**Published:** 2025-05-02

**Authors:** Jie-Hong Ji, Hui-Yuan Wu, Yi-Xin Gao, Chen-Yang Shen, Zi-Wen Yang, Kenneth B. Storey, Dan-Na Yu, Jia-Yong Zhang

**Affiliations:** 1 College of Life Sciences, Zhejiang Normal University, Jinhua 321004, China; 2 Department of Biology, Carleton University, Ottawa, ON K1S 5B6, Canada; 3 Key Lab of Wildlife Biotechnology, Conservation and Utilization of Zhejiang Province, Zhejiang Normal University, Jinhua 321004, China

**Keywords:** mitogenomes, Chilopoda, cryptic species, phylogeny, divergence time

## Abstract

As one of the four primary evolutionary groups within myriapods, centipedes (Chilopoda) comprise approximately 3150 valid species. Recent molecular studies have begun to elucidate the phylogeny and time to divergence in Chilopoda; yet, identifying scutigeromorphs at the species level remains a notoriously challenging task. In this study, we obtained seven new complete mitogenomes of *Thereuopoda clunifera* (Wood, 1862) to investigate the phylogeny and divergence times of Chilopoda. Both maximum likelihood (ML) and Bayesian inference (BI) analyses recovered the relationship of (Scutigeromorpha + (Scolopendromorpha + (Lithobiomorpha + Geophilomorpha))). For Scutigeromorpha, seven newly sequenced mitogenomes of *T. clunifera* were divided into four distinct clades. Divergence time estimates suggest that the basal split of Chilopoda occurred during the Middle Ordovician period, with the origins of Scolopendromorpha, Lithobiomorpha, and Geophilomorpha dating to the Devonian period. Factors such as warm climates, coevolution between predator and prey, and the rifting of the Hainan Island may have driven the diversification of Scutigeromorpha. Based on genetic distance, the delimitation of molecular species, phylogenetic relationships, and divergence time analyses, we identified three cryptic species that existed within *T. clunifera*. This exceptionally high degree of hidden diversity can be ascribed to the morphological stasis that has occurred since the Paleozoic era and taxonomic impediment.

## Simple Summary

Despite extensive research, the phylogeny and divergence times of Chilopoda (centipedes) remain subjects of ongoing debate. In this study, we acquired seven new complete mitogenomes of *Thereuopoda clunifera* (Wood, 1862) to elucidate the phylogenetic relationships and estimate divergence times within Chilopoda. Through the comprehensive analysis of genetic distances, molecular species delimitation, phylogenetic relationships, and divergence times, we identified the presence of three cryptic species within *T. clunifera*.

## 1. Introduction

As an ancient group of soil predators, centipedes (Chilopoda) comprise approximately 3150 valid species distributed across five extant orders: Scutigeromorpha, Lithobiomorpha, Craterostigmomorpha, Scolopendromorpha, and Geophilomorpha [1]. The phylogenetic relationships among these five orders have been a subject of active debate in recent years. The classical debate has centered on whether the most fundamental division within Chilopoda is between Anamorpha (Scutigeromorpha + Lithobiomorpha) and Epimorpha (Scolopendromorpha + Geophilomorpha) based on developmental patterns or, alternatively, between Notostigmophora (Scutigeromorpha) and Pleurostigmophora (the remaining four extant orders) based on the position of spiracles [2]. Most traditional morphological analyses have supported the Pleurostigmophora hypothesis, and the widely accepted consensus on the relationships among the five extant orders of Chilopoda is as follows: (Scutigeromorpha + (Lithobiomorpha + (Craterostigmomorpha + (Scolopendromorpha + Geophilomorpha)))) [2,3,4,5,6]. Molecular systematic research on centipedes began in the late 1990s. The early analyses of ribosomal genes demonstrated strong congruence with morphological data [7]. When two mitochondrial genes were incorporated into these analyses, the resulting phylogenetic framework remained largely stable to parameter variations, and the clade of (Craterostigmomorpha + (Scolopendromorpha + Geophilomorpha)) was designated as Phylactometria [8]. A topology based on three nuclear protein-coding genes revealed a significant inconsistency with morphological expectations, supporting the basal placement of Craterostigmomorpha and the clade of (Scutigeromorpha + Scolopendromorpha) [9]. An analysis incorporating a substantially large number of nuclear protein-coding genes (62 genes) within a broader arthropod phylogeny generally agreed with morphological data but still presented conflicts on the position of Craterostigmomorpha, although this analysis lacked data from Geophilomorpha [10]. Molecular phylogenetic trees, using a combination of nuclear ribosomal and mitochondrial genes, revealed an alliance between Lithobiomorpha and Scolopendromorpha, whereas the monophyly of Phylactometria and Epimorpha were in doubt [11]. Transcriptomic datasets largely recovered the morphological scheme except for the positions of Craterostigmomorpha and Lithobiomorpha [12,13,14,15]. The clade (Lithobiomorpha + (Scolopendromorpha + Geophilomorpha)) was formalized as the following new taxon: Amalpighiata. Furthermore, certain unorthodox groupings, such as the association between Lithobiomorpha and Geophilomorpha, were recovered under specific analytical conditions in transcriptomic analysis and were similarly observed in mitogenomic studies [12,13,14,16,17,18,19,20,21].

Scutigeromorpha, commonly known as a house centipede, is the most distinctive order of centipedes, comprising approximately 95 valid species [22]. The order is characterized by its unique features, including extremely long legs, dorsally positioned spiracles, and compound eyes, which distinguish it from other centipedes [23]. Scutigeromorpha comprises three families: Scutigerinidae, Pselliodidae, and Scutigeridae. The family Scutigeridae includes two subfamilies: Scutigerinae and Thereuoneminae [22]. The phylogeny of Scutigeromorpha has garnered renewed attention recently following an approximately 100-year hiatus. All phylogenetic analyses have consistently found that the three families (Pselliodidae, Scutigerinidae, and Scutigeridae) are monophyletic, with Pselliodidae identified as the sister group to the two other families [24,25,26,27,28]. However, species delimitation within Scutigeromorpha continues to present significant challenges [29,30]. Historically, a prolonged phase of fine species-level splitting by early taxonomists such as Verhoeff and Chamberlin has been followed by widespread synonymy in the 1970s when a polymorphic species concept was adopted. For example, *Thereuopoda longicornis* (Fabricius, 1793), distributed from India to Japan and Australia, has accumulated 26 junior subjective synonyms [31]. This phenomenon may indicate that morphospecies can mask true diversity by concealing the presence of cryptic species. Although there has been considerable debate regarding the best approach to describe this concept, cryptic species are generally defined as morphologically and physiologically similar taxa that are on different evolutionary trajectories [32]. Cryptic species within Scutigeromorpha have repeatedly emerged with the incorporation of molecular data. Yang et al. [18] suggested the presence of cryptic species in *Thereuonema tuberculata* (Wood, 1862) through the analysis of complete mitogenomes. Manivannan et al. [27], employing two molecular species delimitation methods, identified at least 11 putative species within the Peninsular Indian Plate and the Andaman Islands. Edgecombe and Giribet [25] discovered significant genetic diversity within *Scutigera coleoptrata* (Linnaeus, 1758) through the analysis of a few molecular markers.

Estimating the divergence times of centipede lineages using fossil-calibrated dating has become a focus of several molecular studies aimed at elucidating deep branching at the ordinal and familial levels [1]. The fossil record of centipedes is highly incomplete due to their lightly sclerotized cuticle and predominantly litter- and soil-dwelling habits. Studies incorporating Palaeozoic fossils have yielded dated schemes suggesting that the origins of centipede orders occurred during the Palaeozoic era [11,12,13,14,15]. For Scutigeromorpha, the three extant families are characterized by ancient stem groups dating back to the Devonian–Permian periods, whereas their crown-group diversifications are believed to have occurred later, during the Triassic–Jurassic periods.

The typical arthropod mitogenome is a compact circular molecule comprising 13 protein-coding genes (PCGs), 22 transfer RNA (tRNA) genes, 2 ribosomal RNA (rRNA) genes, and a noncoding region (the control region, CR) [33,34]. Characterized by maternal inheritance, a high evolutionary rate, and the absence of recombination, the mitogenome has become a valuable tool for analyzing phylogenetic relationships and detecting cryptic species [18,35,36,37,38,39]. Currently, *T. clunifera*, which has accumulated numerous junior subjective synonyms, has been recorded in China and Japan. However, it has received little attention since Würmli’s work [31]. Therefore, we hypothesize that cryptic species may exist within *T. clunifera*. In this study, we generated seven complete mitogenomes of *T. clunifera* from seven distinct locations. Based on these data, our objectives were as follows: (1) compare the characteristics of the seven mitogenomes of *T. clunifera*; (2) discuss the phylogenetic relationship of Chilopoda; (3) estimate the divergence times within Chilopoda; and (4) explore the potential presence of cryptic species within *T. clunifera*.

## 2. Materials and Methods

### 2.1. Taxon Collection and DNA Extraction

Seven specimens of *T. clunifera* were collected by the button tube method from seven locations in China between 2020 and 2024. Detailed information regarding the specimens is shown in Table 1. All specimens were preserved in 100% ethanol at −20 °C in the Zhang laboratory, College of Life Sciences, Zhejiang Normal University, China. Morphological features, including the head capsule, antennae, mandibles, first and second maxillae, forcipules, tergal plates, legs, sternites, gonopods, subanal plates, and telsons, were examined using an Olympus SZX16 stereomicroscope (Olympus Corporation, Tokyo, Japan). Specimen identification was conducted utilizing the taxonomic key published by Edgecombe [22]. Following the morphological examination, genomic DNA was extracted from the left forelegs of each specimen through the Ezup Column Animal Genomic DNA Purification Kit (Sangon Biotech Company, Shanghai, China).

### 2.2. Mitogenome Generation and Sequence Analyses

Genomic DNA from seven specimens was sequenced through next-generation sequencing by BGI Tech. Inc. (Shenzhen, China). The sequencing was performed on the Illumina HiSeq 2000 platform with 150 bp paired-end reads. The quality of raw data was assessed using FastQC v.0.11.6. Clean data were then used to assemble the mitogenomes via NOVOPlasty v.4.2 [40] and GetOrganelle v.1.7.1 [41]. The tRNA genes were identified using MITOS2 available on the Galaxy platform (https://usegalaxy.eu, accessed on 31 October 2024) [42] and ARWEN v.1.2.3 (http://130.235.244.92/ARWEN/index.html, accessed on 31 October 2024) [43]. PCGs and rRNA genes were annotated with reference to three mitogenomes from the NCBI: *S. coleoptrata* (AJ507061), *T. clunifera* (OL436141), and *T. tuberculata* (ON939554) [20,44]. MEGA v.11 [45] was used to verify the correct translation of PCGs into amino acids. The mitogenomes were visualized using Proksee (https://proksee.ca, accessed on 14 November 2024) [46]. The secondary structure of tRNA was depicted by Forna (http://rna.tbi.univie.ac.at/forna/, accessed on 14 November 2024) [47]. PhyloSuite v.1.2.2 [48] was utilized to calculate relative synonymous codon usage (RSCU), AT content, AT-skew, and GC-skew. The formula for calculating AT-skew and GC-skew are as follows: AT-skew = (A − T)/(A + T), and GC-skew = (G − C)/(G + C).

### 2.3. Species Delimitation

To investigate the potential existence of cryptic species within *T. clunifera*, two molecular species delimitation methods—Assemble Species by Automatic Partitioning (ASAP) [49] and Bayesian Poisson Tree Processes (bPTP) [50]—were employed using 13 concatenated PCGs. ASAP analysis was conducted online (https://bioinfo.mnhn.fr/abi/public/asap/asapweb.html#, accessed on 27 November 2024) using the Kimura (K80) transition/transversion (Ts/Tv) ratio of 2.0 and default parameters. The bPTP analysis was performed online (https://species.h-its.org/ptp/, accessed on 27 November 2024) with the following parameter settings: No. MCMC generations = 500,000; thinning = 100; burn-in = 0.1; and seed = 123. The phylogenetic tree generated by MrBayes v.3.2.7a [51] was required for bPTP analysis. The calculation of genetic distances was performed in MEGA v.11 using the Kimura 2-parameter model [45]. Operational taxonomic units (OTUs) were used to represent species identified through molecular analysis [52].

### 2.4. Dataset Selection and Phylogenetic Analyses

To investigate the phylogeny of Chilopoda, we utilized 25 complete mitogenomes, comprising 7 newly obtained mitogenomes and 18 mitogenomes retrieved from the NCBI database [18,19,20,44,53,54,55,56,57,58]. *Anaulaciulus koreanus* (Verhoeff, 1937) and *Spirobolus bungii* Brandt, 1833, were selected as outgroups for constructing the phylogenetic trees [58]. Detailed information regarding all mitogenomes utilized in this study is presented in Table S1.

The 13 PCGs were extracted using PhyloSuite v.1.2.2 and aligned with MAFFT v.7.475 [59]. Conserved regions were identified using Gblocks v.0.91b [60]. A nucleotide dataset for phylogenetic analyses was constructed by concatenating the 13 PCGs using PhyloSuite v.1.2.2. PartitionFinder v.2.2.1 [61] was employed to partition the nucleotide dataset of 13 PCGs and select the best-fitting models for each partition (Table S2). The maximum likelihood (ML) tree was constructed in RaxML v.8.2.0 [62] with 1000 bootstrap replicates. The Bayesian inference (BI) tree was reconstructed in MrBayes v.3.2.7a [51] with a run of 10 million generations, sampling every 1000 generations, and a burn-in value of 25%. Tree visualization and editing were performed using FigTree v.1.4.4 [63].

### 2.5. Divergence Time Estimation

To assess the divergence times within Chilopoda, we selected five fossils for this study, with their age estimates derived from previous studies [13,64,65,66,67,68,69]. Detailed information regarding fossils is provided in Table 2. Divergence times were estimated using MCMCTree in PAML v.4.8 [70], employing the topology of the ML tree. A root age of 495 Mya was assigned, marking the divergence of Chilopoda [12]. Initially, Baseml was employed to calculate the base substitution rates, and the gradient and Hessian of the branch lengths were estimated using the maximum likelihood method. Subsequently, the approximate likelihood method (usedata = 2) was utilized to estimate divergence times. The parameter settings of MCMCTree were as follows: burn-in = 400,000; sample frequency = 10; and the number of samples = 100,000. Convergence diagnostics were assessed using Trace v.1.7.1 [71], with convergence deemed satisfactory when all effective sample size (ESS) values exceeded 200. The visualization of results was accomplished using FigTree v.1.4.4 [63].

## 3. Results

### 3.1. Composition and Organization of Seven Mitogenomes of T. clunifera

**Figure 1:**
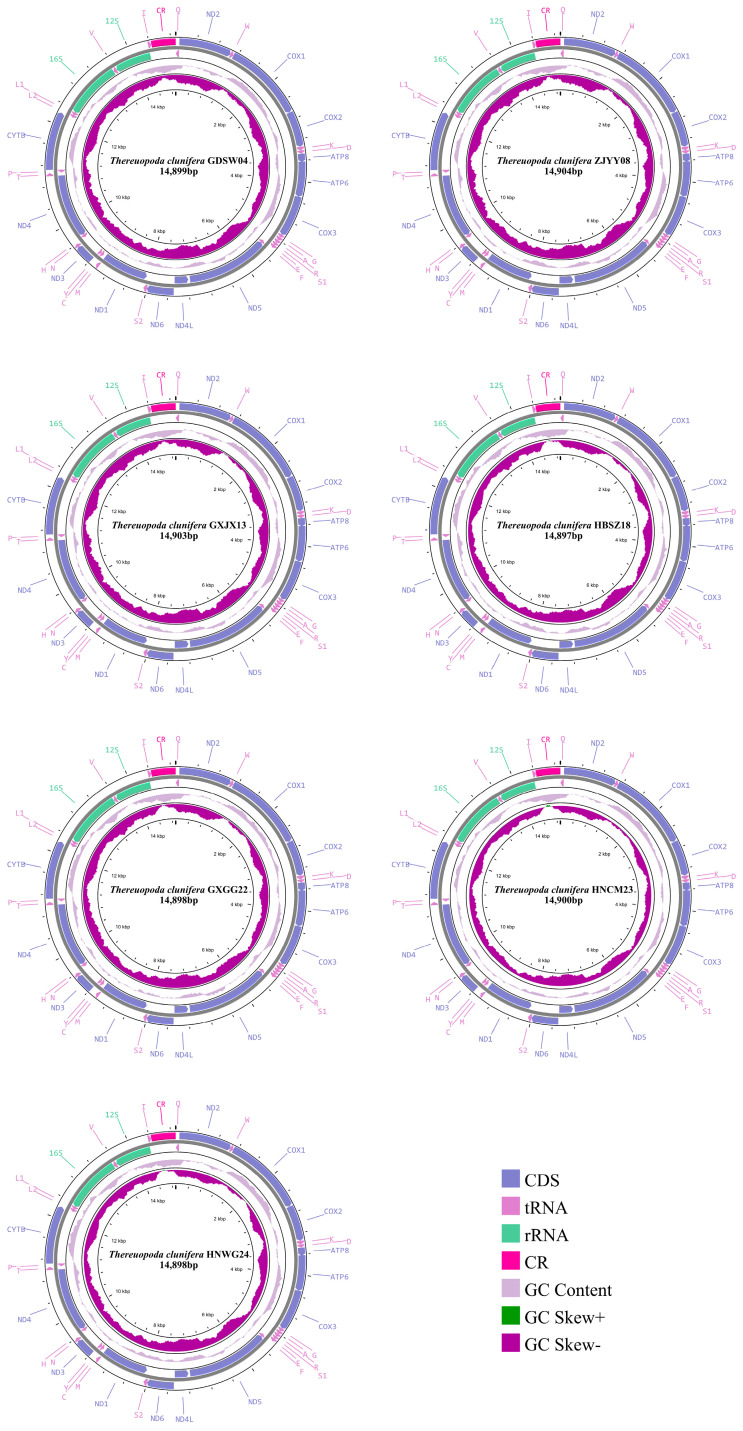
Circular maps of the seven mitogenomes of *T. clunifera*. Genes in the outer circle are encoded on the heavy strand, whereas those in the inner circle are encoded on the light strand. The GC content and GC-skew are depicted as the deviation from the average value of the entire sequence.

Seven complete mitogenomes of *T. clunifera* were sequenced and deposited in GenBank. The lengths of these mitogenomes ranged from 14,897 bp to 14,904 bp (Figure 1). Each mitogenome contained the typical 37 mitochondrial genes. All seven mitogenomes exhibited an identical gene order. The positions of the mitogenome features are detailed in Table S3. Among the 37 genes, 22 genes (ND2, W, COX1, COX2, K, D, ATP8, ATP6, COX3, G, A, R, S1, E, ND6, S2, M, ND3, N, T, CYTB, I) were located on the heavy strand, whereas 15 genes (Q, F, C, Y, H, P, L2, L1, V, ND5, ND4L, ND1, ND4, 16S rRNA and 12S rRNA) were located on the light strand. Some intergenic regions and overlaps were observed in all seven mitogenomes of *T. clunifera*. Specifically, the mitogenomes of *T. clunifera* GDSW04, *T. clunifera* ZJYY08, *T. clunifera* GXJX13, *T. clunifera* HBSZ18, *T. clunifera* HNWG24, *T. clunifera* GXGG22, and *T. clunifera* HNCM23 contained 15, 15, 15, 16, 16, 15, and 16 overlaps, respectively. Most of the overlaps were identical, whereas trnA-trnR and trnK-trnD were absent in *T. clunifera* GDSW04/ZJYY08/GXJX13 and *T. clunifera* GXGG22, respectively. Among the overlapping genes, tRNA genes were the most common, with the exception of the protein-encoding genes ATP8-ATP6 and ATP6-COX3. The number of intergenic regions varied among the mitogenomes: *T. clunifera* GDSW04, ZJYY08, GXJX13, HBSZ18, and HNWG24 each contained eight intergenic regions, whereas *T. clunifera* GXGG22 and HNCM23 each had nine intergenic regions. Overlaps between adjacent genes ranged from 1 bp to 15 bp, whereas intergenic regions varied from 1 bp to 34 bp. An intergenic region between the ND5 and trnF genes was present exclusively in *T. clunifera* GXGG22 and *T. clunifera* HNCM23. Overlaps between adjacent genes ranged from 1 bp to 15 bp, whereas intergenic regions varied from 1 bp to 34 bp. The AT content, AT-skew, and GC-skew of each mitogenome are summarized in Table S4. A high AT content was observed across the seven mitogenomes, with values ranging from 68.1% (*T. clunifera* GXGG22) to 69.8% (*T. clunifera* HBSZ18 and *T. clunifera* HNWG24). Specifically, the AT content for each mitogenome was as follows: 69.3% (*T. clunifera* GDSW04), 69.5% (*T. clunifera* ZJYY08), 69.6% (*T. clunifera* GXJX13), 69.8% (*T. clunifera* HBSZ18 and *T. clunifera* HNWG24), 68.1% (*T. clunifera* GXGG22) and 69.0% (*T. clunifera* HNCM23). The AT-skew values for the seven mitogenomes were 0.037, 0.03, 0.035, 0.016, 0.014, 0.043, and 0.035, respectively. Meanwhile, the GC-skew values were consistently negative, ranging from −0.299 to −0.330.

**Figure 2:**
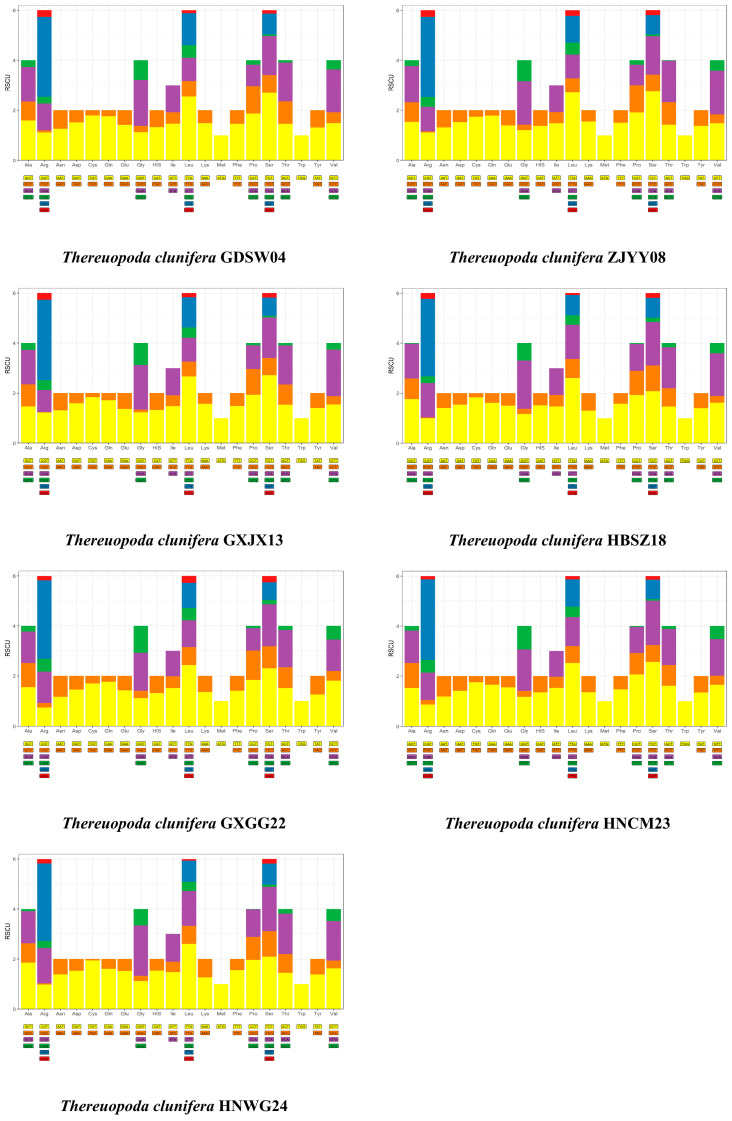
Histograms of the relative synonymous codon usage (RSCU) for the seven mitogenomes. The X-axis represents amino acids and their corresponding codons, whereas the Y-axis indicates RSCU values.

The total length of the 13 PCGs ranged from 11,085 bp to 11,097 bp (Table S4). PCGs on the heavy strand showed a negative AT-skew and GC-skew, whereas those on the light strand showed a negative AT-skew and positive GC-skew. The AT content of PCGs on the light strand was consistently higher than that of PCGs on the heavy strand across all mitogenomes. The predicted start codons for the 13 PCGs were predominantly ATN (ATA/ATG/ATC/ATT), with the exception of the COX1 gene that utilized TTG as its start codon. Regarding stop codons, most PCGs employed complete stop codons (TAA or TAG), whereas an incomplete stop codon (T) was observed in five PCGs: COX2, COX3, ND5, ND3, and ND4. The RSCU of the seven mitogenomes is illustrated in Figure 2. Among the 62 codons, TTT (Phe), ATT (Ile), and TTA (Leu) were the most frequently used, with frequencies exceeding 222 occurrences. Conversely, codons with G or C in the third position were less infrequently utilized, such as TGC (Cys), ACG (Thr), and TCG (Ser).

The total length of the 22 tRNA genes ranged from 1386 bp to 1388 bp (Table S4). Most tRNA genes contained four arms and folded into the typical cloverleaf structure, with the exception of trnS1, which lacked the DHU arm (Figure S1). Mismatches were observed in tRNA genes (Figure S1). Specifically, mismatches in the AA arm included T-T in trnD (*T. clunifera* GDSW04, ZJYY08, GXJX13, GXGG22, and HNCM23), T-C and T-T in trnE (*T. clunifera* GDSW04, ZJYY08, and GXJX13), and T-T in trnN (*T. clunifera* HBSZ18 and HNWG24). Additionally, two mismatches (T-T and T-C) were observed in trnL1 across all seven mitogenomes. For the T arm, mismatches included T-T in trnW in all seven mitogenomes as well as G-G in trnF (*T. clunifera* GXGG22), A-G in trnF (*T. clunifera* HNCM23), and A-G in trnY (*T. clunifera* HBSZ18 and *T. clunifera* HNWG24).

The 16S rRNA gene was positioned between trnL1 and trnV, with lengths of 1188 bp (*T. clunifera* GDSW04), 1190 bp (*T. clunifera* ZJYY08), 1189 bp (*T. clunifera* GXJX13), 1184 bp (*T. clunifera* HBSZ18 and HNWG24), 1188 bp (*T. clunifera* GXGG22), and 1187 bp (*T. clunifera* HNCM23). The 12S rRNA gene was located between trnV and trnI, with lengths of 762 bp (*T. clunifera* GDSW04), 763 bp (*T. clunifera* ZJYY08 and GXJX13), 764 bp (*T. clunifera* HBSZ18, HNWG24, and HNCM23), and 761 bp (*T. clunifera* GXGG22). The AT content of rRNA in these seven mitogenomes ranged from 71.2% to 72.4%, with specific values given as follows: 71.9% (*T. clunifera* GDSW04), 72.4% (*T. clunifera* ZJYY08), 72.1% (*T. clunifera* GXJX13), 71.7% (*T. clunifera* HBSZ18), 72.0% (*T. clunifera* HNWG24), 71.2% (*T. clunifera* GXGG22), and 71.5% (*T. clunifera* HNCM23). The control region was located between the trnI and trnQ genes, ranging from 461 bp to 463 bp.

### 3.2. Species Delimitation

All seven specimens exhibited the following morphological features: the anterior projection of cephalic sutures with divergent posterior parts, borders of tergites VI and VII adorned with heavy spines forming a saw-like fringe, strongly vaulted stoma saddles, elongated stomata, and tergites with evenly rounded posterior borders. Based on these morphological characteristics, all seven specimens were identified as *T. clunifera*. For molecular species delimitation analyses, both ASAP and bPTP methods identified four operational taxonomic units (OTUs) within *T. clunifera* in the most strongly supported partition (Figure S2 and Table S5). Specifically, *T. clunifera* OL436141, *T. clunifera* GDSW04, *T. clunifera* ZJYY08, and *T. clunifera* GXJX13 were grouped into a single OTU. Additionally, *T. clunifera* HBSZ18 and *T. clunifera* HNWG24 formed another single OTU, whereas *T. clunifera* GXGG22 and *T. clunifera* HNCM23 each constituted a separate OTU.

The genetic distances of *T. clunifera* based on the complete mitogenome are presented in Table 3. The overall genetic distance ranged from 0.1% to 18.7%. When the four OTUs delineated by the ASAP and bPTP methods were treated as species entities, the intraspecific genetic distances were significantly lower, ranging from 0.1% to 3.6%, whereas the interspecific genetic distance ranged from 8.9% to 18.7%. The lowest interspecific distance was observed between *T. clunifera* GXGG22 and *T. clunifera* HNCM23 at 8.9%.

### 3.3. Phylogenetic Analyses

**Figure 3:**
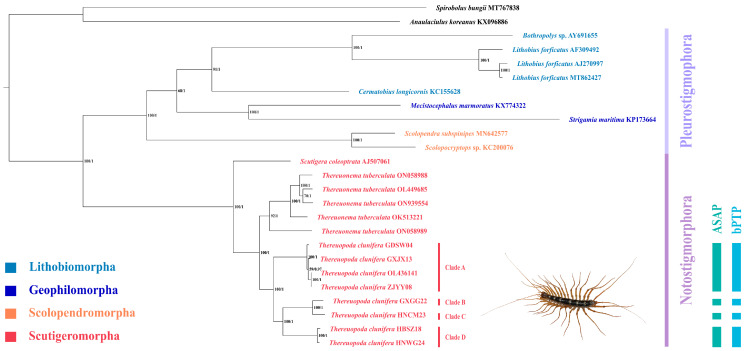
Phylogenetic relationships within Chilopoda were inferred from ML and BI analyses based on the 13 PCGs. Bootstrap values are shown on the left side of the branches, while posterior probabilities are shown on the right side. The columns on the right display the results of two molecular species delimitation methods: ASAP and bPTP.

The ML and BI trees exhibited a completely consistent topology (Figure 3). Within Chilopoda, two primary clades were identified: one comprising the single-order Scutigeromorpha and the other including the remaining three orders (Scolopendromorpha + (Lithobiomorpha + Geophilomorpha)). Both ML and BI analyses strongly supported the monophyly of the four orders. Scutigeromorpha was identified as the earliest diverging lineage within Chilopoda, whereas Scolopendromorpha was recovered as the sister clade (Lithobiomorpha + Geophilomorpha).

For Scutigeromorpha, the monophyly of the family Scutigeridae and subfamily Thereuoneminae was recovered. However, the monophyly of the subfamily Scutigerinae requires further investigation due to the limited availability of mitogenomes. The clade of (*Thereuopoda* Verhoeff, 1904 + *Thereuonema* Verhoeff, 1904) was identified as the sister clade to *Scutigera* Lamarck, 1801. At the species level, four well-supported clades were identified within *T. clunifera* as follows: Clade A (*T. clunifera* GDSW04, *T. clunifera* ZJYY08, *T. clunifera* GXJX13, and *T. clunifera* OL436141), Clade B (*T. clunifera* GXGG22), Clade C (*T. clunifera* HNCM23), and Clade D (*T. clunifera* HBSZ18 and *T. clunifera* HNWG24). The phylogenetic relationship was as follows: (Clade A + (Clade D + (Clade B + Clade C))).

### 3.4. Divergence Time Estimation

**Figure 4:**
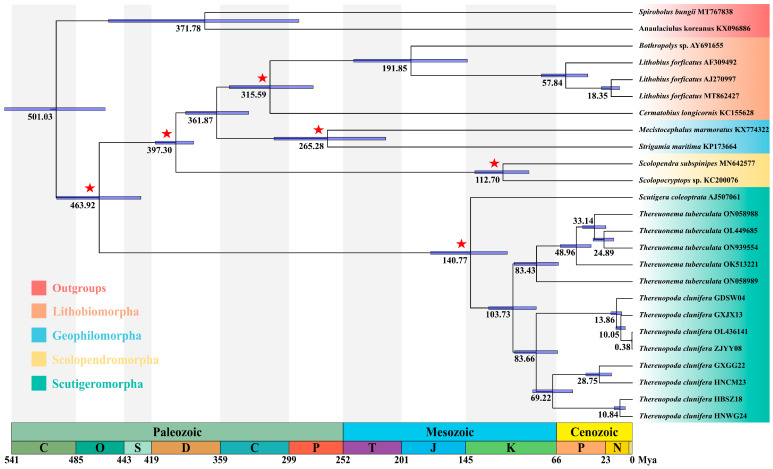
The chronogram of Chilopoda was inferred using MCMCTree, with the 95% highest posterior density (HPD) intervals indicated by bars. Stars on the nodes denote the placement of the fossil calibrations. Median ages are provided below each node.

The dated phylogeny of Chilopoda based on the 13 PCGs reflects the divergence between Notostigmophora and Pleurostigmophora in the Middle Ordovician period (463.92 Mya, 95% HPD: 426.36–500.30 Mya), the divergence between Scolopendromorpha and (Lithobiomorpha + Geophilomorpha) in the Lower Devonian period (397.30 Mya, 95% HPD: 381.48–415.13 Mya), and the divergence between Lithobiomorpha and Geophilomorpha in the Upper Devonian period (361.87 Mya, 95% HPD: 334.55–389.06 Mya) (Figure 4). The diversification of Scutigeridae occurred in the Lower Cretaceous period (140.77 Mya, 95% HPD: 108.95–175.91 Mya), whereas the clade of (*Thereuopoda* + *Thereuonema*) began to diversify in the Lower Cretaceous period (103.73 Mya, 95% HPD: 83.40–124.82 Mya). During the Upper Cretaceous period, *Thereuopoda* and *Thereuonema* began diverging at 83.66 Mya (95% HPD: 65.44–102.41 Mya) and 83.43 Mya (95% HPD: 64.61–102.95 Mya), respectively. Within *T. clunifera*, the estimated divergence times for the basal split of four identified clades from the phylogenetic analysis were as follows: Clade A at 13.86 Mya (95% HPD: 9.37–18.94 Mya), Clade B at 28.75 Mya (95% HPD: 17.99–40.28 Mya), Clade C at 28.75 Mya (95% HPD: 17.99–40.28 Mya), and Clade D at 10.84 Mya (95% HPD: 6.25–15.95 Mya).

## 4. Discussion

### 4.1. Structure of Seven Mitogenomes of T. clunifera

Seven newly sequenced mitogenomes of *T. clunifera* contained the 37 genes usually found in Arthropoda mitogenomes [33]. The genetic order of these seven mitogenomes was identical to those of other available mitogenomes within Scutigeromorpha, which is unique among mitogenomes determined for the Arthropoda [18,20,44]. A comparison with the primitive gene order of *Limulus polyphemus* (Linnaeus, 1758) revealed at least ten translocation events involving six tRNAs genes (trnN, trnS2, trnI, trnM, trnC, and trnY) and four PCGs (ND3, ND4L, ND6, and ND1) [44,72]. The unique arrangement observed in *T. clunifera* can be explained by the tandem duplication–random loss (TDRL) model [20,33,44]. The conserved tandem gene cluster ATP8-ATP6, commonly observed in mitogenomes, was also present in *T. clunifera* [73]. However, two well-established sets of tandem genes, ND4-ND4L, and ND6-CYTB, that are commonly found in most Chilopoda mitogenomes were disrupted in *T. clunifera* [19,33]. Similar characteristics have been observed in other mitogenomes, such as that of *Tigriopus japonicus* Mori, 1932 [74].

All mitogenomes exhibited a positive AT-skew and a negative GC-skew, which may be attributed to the asymmetric mutation processes observed during replication [75]. The start codons predicted for the 13 PCGs in the seven mitogenomes of *T. clunifera* were predominantly conventional start codons ATN and TTG, whereas unusual start codons TTA and TAT were identified in *S. coleoptrata* and *T. tuberculata*, respectively [18,44,73]. Most PCGs were terminated with the complete stop codons TAA or TAG, except for five PCGs (COX2, COX3, ND3, ND4 and ND5), which ended with the incomplete stop codon T, likely completed by post-transcriptional polyadenylation [76]. The majority of tRNA genes exhibited the typical cloverleaf structure. However, the DHU arm was absent in trnS1, which is a characteristic commonly observed in metazoan mitogenomes [18,20,44,53]. Compared with the normal structures, these deficiencies reduced translational activity [77]. Additionally, the mismatched base pairs in some tRNA genes might also affect aminoacylation and translation [78]. The mismatches in the AA arm were probably corrected through a peculiar process of editing that has been verified in *Lithobius forficatus* (Linnaeus, 1758) [53].

### 4.2. Phylogenetic Relationship of Chilopoda

Due to the limited availability of mitogenomes with credible annotations of Chilopoda, only 23 mitogenomes of Chilopoda and 2 mitogenomes of Diplopoda as outgroups were used to construct the ML and BI trees, highlighting the need to acquire additional mitogenomes in this group. In our analyses, both ML and BI trees strongly supported the monophyly of the four orders. Additionally, Scutigeromorpha was recovered as the sister group to the other three orders. This result provides further evidence for the Pleurostigmophora hypothesis, which posits that Chilopoda is divided into two subclasses, Notostigmophora and Pleurostigmophora, based on the position of spiracles [79]. The Pleurostigmophora hypothesis has been accepted by most studies [2,7,8,11,12,13,14,15]. However, some transcriptomic analyses have placed Craterostigmomorpha as a sister group to Scutigeromorpha, a relationship that has not been supported by morphological evidence [12,13,16]. Within Pleurostigmophora, we observed the following relationship (Scolopendromorpha + (Lithobiomorpha + Geophilomorpha)), which has also been reported in studies using mitogenomes or transcriptomic data under certain analytical conditions. This finding contradicts the classical relationship of (Geophilomorpha + Scolopendromorpha) [12,13,14,16,17,18,19,20,21]. In terms of morphology, the former scheme was identified in a non-numerical phylogenetic study based on a single feature of sperm structure. However, perspectives from development, behavior, external morphology, and internal anatomy (including eight autapomorphies described by Edgecombe) align with the classical scheme [80,81]. Furthermore, one of the most contentious issues in current research is the phylogenetic position of Craterostigmomorpha [1]. The Phylactometria hypothesis, named for the maternal care of eggs and hatchlings, is supported by morphology and behavioral evidence. In contrast, the competing Amalpighiata hypothesis, which posits an exchange in the positions of Craterostigmomorpha and Lithobiomorpha, is grounded in phylogenomic data [8,14]. However, the limited availability of mitogenomes for Craterostigmomorpha has hindered further exploration of its phylogenetic position. For Scutigeromorpha, our analysis confirmed the monophyly of the family Scutigeridae, which is consistent with all previous phylogenetic studies to date [24,25,26,27,28]. The monophyly of the two subfamilies Scutigerinae and Thereuoneminae remained uncertain due to the poor internal resolution of Scutigeridae in some studies, although the monophyly of Thereuoneminae was supported in this study [26,28]. Within *T. clunifera*, eight mitogenomes are clustered into four distinct clades, suggesting that these clades may represent different species. Future research will include additional mitogenomes to further elucidate the phylogeny of centipedes and the phylogenetic relationships within Scutigeromorpha.

### 4.3. Divergence Time of Chilopoda

The basal split of Chilopoda was dated to the Middle Ordovician period (463.92 Mya), which is not significantly earlier than the first appearance of the crown group chilopod fossils, *Crussolum*, in the Late Silurian period [82]. This result is generally consistent with the timing observed in previous studies [11,12,13,14,15]. The origins of Scolopendromorpha, Lithobiomorpha, and Geophilomorpha were estimated to have occurred during the Devonian period, aligning with the timing inferred from transcriptomic data [12,13,14], although these estimates are slightly younger than those provided by Murienne et al. using a limited number of molecular markers [11]. The earliest diversification of the family Scutigeridae is estimated to have occurred in the Upper Triassic or Lower Jurassic periods [26,27], but in this study, the estimate was considerably younger, dating to approximately 140.77 Mya, which may be attributed to the density of the taxon sampling. Additionally, the diversification of Scutigeridae during the Lower Cretaceous period supports the hypothesis that the Crato fossil *Fulmenocursor tenax* belongs to the extant family Scutigeridae, as suggested by its morphological characteristics [69].

The Cretaceous period exemplifies a greenhouse climate in Earth’s history, characterized by prolonged warmth lasting until approximately 70 Mya, attributed to elevated atmospheric CO_2_ concentrations [83]. Our findings indicate that the genera of scutigeromorphs originated during the Cretaceous period. The warming climate appeared to have significantly influenced the diversification of scutigeromorphs, as evidenced by similar patterns observed in mayflies, spiders, and salamanders [38,84,85,86]. Furthermore, scutigeromorphs exhibit a broad dietary spectrum, encompassing various spiders and insects such as moths, butterflies, cockroaches, and termites. These taxa underwent substantial diversification during the Cretaceous period [84,87,88,89,90]. Consequently, predator–prey coevolution likely played a crucial role in the radiation and development of scutigeromorphs. Notably, the K-Pg extinction event may have had a limited impact on scutigeromorphs due to their small body size, high starvation tolerance, and effective sheltering capabilities, which could facilitate their survival during extreme climatic events [91].

Historical vicariance events have been identified as a key factor driving the global biogeography of scutigeromorphs [26,27,28]. According to the geological tectonic theory of Southeast Asia, Hainan Island remained connected to northern Vietnam and southwest Guangxi approximately 30 Mya [92,93]. The collision between the Indian Plate and the Eurasian Plate resulted in the southeastward displacement of Hainan Island, which ultimately reached its present location about 15 Mya [94]. The divergence between *T. clunifera* GXGG22 and *T. clunifera* HNCM23 occurred at approximately 28.75 Mya (95% HPD: 17.99–40.28 Mya), coinciding with the rifting event that separated Hainan Island from the mainland. This geological event likely contributed to the divergence between these two groups.

### 4.4. Identification of Cryptic Species

Morphologically, all seven specimens collected from different locations were identified as *T. clunifera* based on their morphological characteristics. However, the genetic distance between these specimens, based on complete mitogenome analysis, ranged from 0.1% to 18.7%, suggesting the presence of cryptic species within *T. clunifera* [25]. This result was also supported by ASAP and bPTP analyses, which identified four OTUs within *T. clunifera*. When taxonomy was based on OTUs, the genetic distances between the four OTUs assigned to *T. clunifera* ranged from 8.9% to 18.7%, whereas intra-OTU genetic distances were much lower, ranging from 0.1% to 3.6%. Wesener et al. found that interspecific genetic distances among *Cryptops* Leach, 1815, species ranged from 13.7% to 22.2%, and three distinct lineages of *Cryptops parisi* Brolemann, 1920, differed by 8.4% to 11.3% from one another, which might represent distinct species [95]. Similarly, Yang et al. discovered that the genetic distances among *T. tuberculata* specimens from four different geographic locations ranged from 7.7% to 15.2%, leading them to hypothesize that these populations could be cryptic species [18]. Edgecombe and Giribet reported that the average *p*-distance within the western Australian *Allothereua* Verhoeff 1905 clade for the COX1 gene was 9.0% compared to 13.3% for the eastern Australia/New Caledonia clade [25]. Based on these findings, we conclude that the species delimitation suggested by ASAP and bPTP analyses accurately represents true species boundaries in *T. clunifera*, comprising a complex of four distinct species. Phylogenetic analyses revealed that eight mitogenomes of *T. clunifera* were assigned to four distinct clades. Furthermore, each of the four clades diverged at least 28.75 Mya, predating the earliest intra-clade (13.86 Mya) diversification. The phylogeny and divergence times underpin the above conclusion.

The unusual extent of hidden diversity within *T. clunifera* can be attributed to extreme morphological conservatism in the modern species and the high level of morphological stasis observed since the Palaeozoic period [26]. This phenomenon may also stem from taxonomic challenges, including the difficulty of capturing specimens and the increasing scarcity of experienced taxonomists specializing in Scutigeromorpha [23]. The World Catalogue of Centipedes, ChiloBase 2.0, listed four taxa in the synonymy of *T. clunifera* [96]. Würmli proposed those synonymies after recognizing the significance of postmaturational molts, which contributed to the trend of adopting polymorphic species [31]. However, geometric morphometric analyses have raised reasonable doubts about the accuracy of the polymorphic species hypothesis [97,98]. It is essential to conduct an integrated taxonomy of *T. clunifera* in the future, and we believe that our findings will drive significant taxonomic revisions of the *T. clunifera* complex.

## 5. Conclusions

Seven mitogenomes of *T. clunifera* were sequenced to investigate the phylogeny and divergence times of Chilopoda. ML and BI analyses revealed that four orders of Chilopoda (Scutigeromorpha, Scolopendromorpha, Geophilomorpha, and Lithobiomorpha) are monophyletic. The Pleurostigmophora hypothesis, which posits that Chilopoda is divided into Notostigmophora and Pleurostigmophora, was supported by these analyses. Scolopendromorpha was identified as the sister clade to the (Lithobiomorpha + Geophilomorpha) group. Divergence time analysis indicated that the diversification of Chilopoda began in the Middle Ordovician period, with Scolopendromorpha, Lithobiomorpha, and Geophilomorpha originating in the Devonian period. Factors such as a warm climate, predator–prey coevolution, and the rifting of the Hainan Island may have driven the diversification of Scutigeromorpha. Based on genetic distance, molecular species delimitation, phylogenetic relationships, and divergence time, we concluded that *T. clunifera* comprises a complex of four cryptic species. Taxonomic impediments and morphological stasis have occurred in in-house centipedes since the Paleozoic period and have likely contributed to the high level of hidden diversity observed.

## Figures and Tables

**Table 1:** Detailed information of seven specimens.

Number	Species	Sampling Localities	Accession No.
ZJYY08	*T. clunifera*	Ningbo, Zhejiang, China	PQ595907
GDSW04	*T. clunifera*	Shanwei, Guangdong, China	PQ595908
GXGG22	*T. clunifera*	Guigang, Guangxi, China	PQ595909
GXJX13	*T. clunifera*	Laibin, Guangxi, China	PQ595910
HBSZ18	*T. clunifera*	Suizhou, Hubei, China	PQ595911
HNCM23	*T. clunifera*	Chengmai, Hainan, China	PQ595912
HNWG24	*T. clunifera*	Pingdingshan, Henan, China	PQ595913

**Table 2:** Fossil species used for dating in this study, along with parameters for MCMCTree and references that provide phylogenetic and age justifications.

Dated Crown Group	Calibration Fossil	Bound for MCMCTree	Phylogenetic Justification	Age Justification
Crown Chilopoda	*Crussolum* sp.	B (4.16, 5.28)	[13]	[68]
Crown Scutigeromorpha	*Fulmenocursor tenax*	B (1.12, 4.16)	[69]	[68]
Crown Pleurostigmophora	*Devonobius delta*	B (3.82, 4.16)	[13]	[68]
Crown Lithobiomorpha	*Henicopidae* sp.	B (0.98, 3.82)	[65]	[65]
Crown Geophilomorpha	*Kachinophilus pereirai*	B (0.98, 3.09)	[66,67]	[68]
Crown Scolopendromorpha	*Cratoraricrus oberlii*	B (1.12, 3.09)	[64]	[68]

**Table 3:** The genetic distance of *T. clunifera* based on the complete mitogenome.

Number	GDSW04	ZJYY08	GXJX13	OL436141	GXGG22	HNCM23	HBSZ18
GDSW04							
ZJYY08	0.036						
GXJX13	0.032	0.033					
OL436141	0.036	0.001	0.033				
GXGG22	0.183	0.184	0.179	0.184			
HNCM23	0.187	0.187	0.185	0.187	0.089		
HBSZ18	0.179	0.178	0.177	0.178	0.180	0.183	
HNWG24	0.179	0.177	0.176	0.177	0.177	0.182	0.033

## Data Availability

Supporting data for this study are available from the National Center for Biotechnology Information (https://www.ncbi.nlm.nih.gov) (accessed on 15 November 2024). The GenBank numbers are PP595907-PP595913.

## Supplementary Materials

The following supporting information can be downloaded at: https://www.mdpi.com/article/10.3390/insects16050486/s1. Figure S1. The secondary structure of tRNAs in seven newly sequenced mitogenomes; Figure S2. Results of species delimitation using ASAP based on the thirteen PCGs; Table S1. Species used to construct the phylogenetic relationships along with GenBank accession numbers; Table S2. Best partitioning scheme and best-fitting models selected by PartitionFinder v.2.2.1; Table S3. Location of features of seven mitogenomes; Table S4. Base composition of 13 PCGs in seven mitogenomes; Table S5. Results of species delimitation using bPTP based on the thirteen PCGs.
