# Supplementary material for: Unusual Genetic Diversity Within Thereuopoda clunifera (Wood, 1862) (Chilopoda: Scutigeromorpha) Revealed by Phylogeny and Divergence Times Using Mitochondrial Genomes
Source: Insects. 2025 May 2;16(5):486. doi: 10.3390/insects16050486 (PMC12112239; doi:10.3390/insects16050486)
Supplement: Supplementary file 1 [file insects-16-00486-s001.zip › Table S1.pdf]

TableS1. Species used to construct the phylogenetic relationships along with GenBank accession numbers.

Class	Order	Family	Species	Length (bp)	GenBank No.
Diplopoda	Julida	Julidae	*Anaulaciulus koreanus*	14,916	KX096886
	Spirobolida	Spirobolidae	*Spirobolus bungii*	14,879	MT767838
Chilopoda	Scutigeromorpha	Scutigeridae	*Scutigera coleoptrata*	14,922	AJ507061
			Thereuonema tuberculata	14,902	ON939554
			Thereuonema tuberculata	14,906	OL449685
			Thereuonema tuberculata	14,909	ON058988
			Thereuonema tuberculata	14,903	ON058989
			Thereuonema tuberculata	14,905	OK513221
			Thereuopoda clunifera	14,898	OL436141
			Thereuopoda clunifera	14,899	PQ595908
			Thereuopoda clunifera	14,904	PQ595907
			Thereuopoda clunifera	14,903	PQ595910
			Thereuopoda clunifera	14,897	PQ595911
			Thereuopoda clunifera	14,898	PQ595909
			Thereuopoda clunifera	14,900	PQ595912
			Thereuopoda clunifera	14,898	PQ595913
	Lithobiomorpha	Lithobiidae	*Lithobius forficatus*	15,695	AF309492
			Lithobius forficatus	15,437	AJ270997
			Lithobius forficatus	15,038	MT862427
		Ethopolyidae	*Bothropolys* sp. SP-2004	15,139	AY691655
		Henicopidae	*Cermatobius longicornis*	16,833	KC155628
	Scolopendromorpha	Scolopendridae	*Scolopendra subspinipes*	14,637	MN642577
		Cryptopidae	*Scolopocryptops* sp. 1 YG-2013	15,119	KC200076
	Geophilomorpha	Linotaeniidae	*Strigamia maritima*	14,983	KP173664

	Mecistocephalidae	*Mecistocephalus marmoratus*	15,279	KX774322
