# Supplementary material for: Unusual Genetic Diversity Within Thereuopoda clunifera (Wood, 1862) (Chilopoda: Scutigeromorpha) Revealed by Phylogeny and Divergence Times Using Mitochondrial Genomes
Source: Insects. 2025 May 2;16(5):486. doi: 10.3390/insects16050486 (PMC12112239; doi:10.3390/insects16050486)
Supplement: Supplementary file 1 [file insects-16-00486-s001.zip › Table S2.pdf]

Table S2. Best partitioning scheme and best-fitting models selected by PartitionFinder v.2.2.1.

Nucleotide sequence alignments		
**Subset**	**Subset partitions**	**Best model**
Partition 1	ND3_codon1, ATP6_codon1, COX2_codon1, COX3_codon1, CYTB_codon1	GTR + I + G
Partition 2	COX2_codon2, COX3_codon2, ATP6_codon2, CYTB_codon2	TVM + G
Partition 3	CYTB_codon3, COX2_codon3, COX1_codon3, COX3_codon3, ATP6_codon3	GTR + I + G
Partition 4	ATP8_codon1, ND2_codon1, ND6_codon1	GTR + I + G
Partition 5	ATP8_codon2, ND3_codon2, ND6_codon2, ND2_codon2	TVM + I + G
Partition 6	ND2_codon3, ND6_codon3, ND3_codon3, ATP8_codon3	TRN + G
Partition 7	COX1_codon1	GTR + I + G
Partition 8	COX1_codon2	TVM + G
Partition 9	ND4_codon1, ND5_codon1, ND4L_codon1, ND1_codon1	GTR + I + G
Partition 10	ND1_codon2, ND4L_codon2, ND5_codon2, ND4_codon2	GTR + I + G
Partition 11	ND1_codon3, ND5_codon3, ND4_codon3, ND4L_codon3	GTR + G
