# Supplementary material for: Unusual Genetic Diversity Within Thereuopoda clunifera (Wood, 1862) (Chilopoda: Scutigeromorpha) Revealed by Phylogeny and Divergence Times Using Mitochondrial Genomes
Source: Insects. 2025 May 2;16(5):486. doi: 10.3390/insects16050486 (PMC12112239; doi:10.3390/insects16050486)
Supplement: Supplementary file 1 [file insects-16-00486-s001.zip › Table S3.pdf]

Table S3. Location of features of seven mitogenomes

A. *Thereuopoda clunifera* GDSW04

Gene	Strand	From	To	Start	Stop
trnQ	L	1	68		
ND2	H	70	1065	ATT	TAG
trnW	H	1064	1126		
COX1	H	1126	2661	TTG	TAA
COX2	H	2665	3343	ATG	T
trnK	H	3345	3414		
trnD	H	3414	3476		
ATP8	H	3477	3632	ATT	TAG
ATP6	H	3626	4300	ATG	TAA
COX3	H	4300	5086	ATG	T
trnG	H	5087	5147		
trnA	H	5147	5211		
trnR	H	5212	5272		
trnS1	H	5273	5330		
trnE	H	5329	5388		
trnF	L	5388	5447		
ND5	L	5448	7155	TTG	T
ND4L	L	7189	7473	ATG	TAA
ND6	H	7489	7998	ATC	TAA
trnS2	H	7997	8065		
ND1	L	8056	9015	ATA	TAA
trnM	H	9001	9063		
trnC	L	9063	9122		
trnY	L	9123	9183		
ND3	H	9185	9536	ATC	T
trnN	H	9537	9599		
trnH	L	9599	9660		
ND4	L	9661	10996	ATG	T
trnT	H	10999	11059		
trnP	L	11057	11117		
CYTB	H	11119	12234	ATG	TAA
trnL2	L	12235	12296		
trnL1	L	12293	12353		
16S rRNA	L	12354	13541		
trnV	L	13542	13612		
12S rRNA	L	13613	14374		
trnI	H	14375	14438		

B. *Thereuopoda clunifera* ZJYY08

Gene	Strand	From	To	Start	Stop
trnQ	L	1	68		
ND2	H	70	1065	ATT	TAG
trnW	H	1064	1126		
COX1	H	1126	2661	TTG	TAA
COX2	H	2665	3343	ATG	T
trnK	H	3345	3414		
trnD	H	3414	3476		
ATP8	H	3477	3632	ATT	TAG
ATP6	H	3626	4300	ATG	TAA
COX3	H	4300	5086	ATG	T
trnG	H	5087	5147		
trnA	H	5147	5211		
trnR	H	5212	5272		
trnS1	H	5273	5330		
trnE	H	5329	5388		
trnF	L	5388	5447		
ND5	L	5448	7155	TTG	T
ND4L	L	7189	7473	ATG	TAA
ND6	H	7484	7999	ATA	TAA
trnS2	H	7998	8066		
ND1	L	8057	9016	ATA	TAA
trnM	H	9002	9064		
trnC	L	9064	9123		
trnY	L	9124	9184		
ND3	H	9186	9537	ATC	T
trnN	H	9538	9600		
trnH	L	9600	9661		
ND4	L	9662	10997	ATG	T
trnT	H	11000	11060		
trnP	L	11058	11118		
CYTB	H	11120	12235	ATG	TAA
trnL2	L	12236	12297		
trnL1	L	12294	12354		
16S rRNA	L	12355	13544		
trnV	L	13545	13615		
12S rRNA	L	13616	14378		
trnI	H	14379	14442		

C. *Thereuopoda clunifera* GXJX13

Gene	Strand	From	To	Start	Stop
trnQ	L	1	68		
ND2	H	70	1065	ATC	TAG
trnW	H	1064	1126		
COX1	H	1126	2661	TTG	TAA
COX2	H	2665	3343	ATG	T
trnK	H	3345	3414		
trnD	H	3414	3476		
ATP8	H	3477	3632	ATT	TAG
ATP6	H	3626	4300	ATG	TAA
COX3	H	4300	5086	ATG	T
trnG	H	5087	5147		
trnA	H	5147	5211		
trnR	H	5212	5272		
trnS1	H	5273	5330		
trnE	H	5329	5388		
trnF	L	5388	5447		
ND5	L	5448	7155	TTG	T
ND4L	L	7189	7473	ATG	TAA
ND6	H	7496	7999	ATC	TAA
trnS2	H	7998	8066		
ND1	L	8057	9016	ATA	TAA
trnM	H	9002	9064		
trnC	L	9064	9123		
trnY	L	9124	9184		
ND3	H	9186	9537	ATC	T
trnN	H	9538	9600		
trnH	L	9600	9661		
ND4	L	9662	10997	ATG	T
trnT	H	11000	11060		
trnP	L	11058	11118		
CYTB	H	11120	12235	ATG	TAA
trnL2	L	12236	12297		
trnL1	L	12294	12354		
16S rRNA	L	12355	13543		
trnV	L	13544	13614		
12S rRNA	L	13615	14377		
trnI	H	14378	14441		

D. *Thereuopoda clunifera* HBSZ18

Gene	Strand	From	To	Start	Stop
trnQ	L	1	68		
ND2	H	70	1065	ATT	TAG
trnW	H	1064	1126		
COX1	H	1126	2661	TTG	TAA
COX2	H	2665	3343	ATG	T
trnK	H	3345	3414		
trnD	H	3414	3475		
ATP8	H	3476	3631	ATT	TAG
ATP6	H	3625	4299	ATG	TAA
COX3	H	4299	5085	ATG	T
trnG	H	5086	5146		
trnA	H	5146	5210		
trnR	H	5210	5270		
trnS1	H	5271	5326		
trnE	H	5325	5386		
trnF	L	5386	5445		
ND5	L	5446	7153	TTG	T
ND4L	L	7186	7470	ATG	TAA
ND6	H	7486	7995	ATT	TAA
trnS2	H	7994	8062		
ND1	L	8053	9012	ATA	TAA
trnM	H	8998	9060		
trnC	L	9060	9119		
trnY	L	9120	9181		
ND3	H	9183	9534	ATT	T
trnN	H	9535	9597		
trnH	L	9597	9659		
ND4	L	9660	10995	ATG	T
trnT	H	10998	11058		
trnP	L	11056	11116		
CYTB	H	11118	12233	ATG	TAA
trnL2	L	12234	12295		
trnL1	L	12292	12352		
16S rRNA	L	12353	13536		
trnV	L	13537	13607		
12S rRNA	L	13608	14371		
trnI	H	14372	14435		

E. *Thereuopoda clunifera* GXGG22

Gene	Strand	From	To	Start	Stop
trnQ	L	1	68		
ND2	H	70	1065	ATT	TAG
trnW	H	1064	1126		
COX1	H	1126	2661	TTG	TAA
COX2	H	2665	3343	ATG	T
trnK	H	3345	3414		
trnD	H	3415	3475		
ATP8	H	3476	3631	ATT	TAG
ATP6	H	3625	4299	ATG	TAA
COX3	H	4299	5085	ATG	T
trnG	H	5086	5147		
trnA	H	5147	5211		
trnR	H	5211	5271		
trnS1	H	5272	5329		
trnE	H	5328	5389		
trnF	L	5389	5448		
ND5	L	5450	7157	TTG	T
ND4L	L	7191	7475	ATG	TAA
ND6	H	7491	8000	ATC	TAA
trnS2	H	7999	8067		
ND1	L	8058	9017	ATA	TAA
trnM	H	9003	9064		
trnC	L	9064	9123		
trnY	L	9124	9184		
ND3	H	9186	9537	ATT	T
trnN	H	9538	9600		
trnH	L	9600	9661		
ND4	L	9662	10997	ATG	T
trnT	H	11000	11061		
trnP	L	11059	11119		
CYTB	H	11121	12236	ATG	TAA
trnL2	L	12237	12298		
trnL1	L	12295	12355		
16S rRNA	L	12356	13543		
trnV	L	13544	13612		
12S rRNA	L	13613	14373		
trnI	H	14374	14437		

F. *Thereuopoda clunifera* HNCM23

Gene	Strand	From	To	Start	Stop
trnQ	L	1	68		
ND2	H	70	1065	ATC	TAG
trnW	H	1064	1126		
COX1	H	1126	2661	TTG	TAA
COX2	H	2665	3343	ATG	T
trnK	H	3345	3414		
trnD	H	3414	3475		
ATP8	H	3476	3631	ATT	TAG
ATP6	H	3625	4299	ATG	TAA
COX3	H	4299	5085	ATG	T
trnG	H	5086	5146		
trnA	H	5146	5210		
trnR	H	5210	5270		
trnS1	H	5271	5328		
trnE	H	5327	5388		
trnF	L	5388	5447		
ND5	L	5449	7156	TTG	T
ND4L	L	7191	7475	ATG	TAA
ND6	H	7491	8000	ATC	TAA
trnS2	H	7999	8067		
ND1	L	8058	9017	ATA	TAA
trnM	H	9003	9064		
trnC	L	9064	9123		
trnY	L	9124	9184		
ND3	H	9186	9537	ATT	T
trnN	H	9538	9600		
trnH	L	9600	9661		
ND4	L	9662	10997	ATG	T
trnT	H	11000	11061		
trnP	L	11059	11119		
CYTB	H	11121	12236	ATG	TAA
trnL2	L	12237	12298		
trnL1	L	12295	12355		
16S rRNA	L	12356	13542		
trnV	L	13543	13611		
12S rRNA	L	13612	14375		
trnI	H	14376	14439		

F. *Thereuopoda clunifera* HNWG24

Gene	Strand	From	To	Start	Stop
trnQ	L	1	68		
ND2	H	70	1065	ATT	TAG
trnW	H	1064	1126		
COX1	H	1126	2661	TTG	TAA
COX2	H	2665	3343	ATG	T
trnK	H	3345	3414		
trnD	H	3414	3475		
ATP8	H	3476	3631	ATT	TAG
ATP6	H	3625	4299	ATG	TAA
COX3	H	4299	5085	ATG	T
trnG	H	5086	5146		
trnA	H	5146	5210		
trnR	H	5210	5270		
trnS1	H	5271	5326		
trnE	H	5325	5386		
trnF	L	5386	5445		
ND5	L	5446	7153	TTG	T
ND4L	L	7186	7470	ATG	TAA
ND6	H	7486	7995	ATA	TAA
trnS2	H	7994	8062		
ND1	L	8053	9012	ATA	TAA
trnM	H	8998	9060		
trnC	L	9060	9119		
trnY	L	9120	9181		
ND3	H	9183	9534	ATT	T
trnN	H	9535	9597		
trnH	L	9597	9659		
ND4	L	9660	10995	ATG	T
trnT	H	10998	11058		
trnP	L	11056	11116		
CYTB	H	11118	12233	ATG	TAA
trnL2	L	12234	12295		
trnL1	L	12292	12352		
16S rRNA	L	12353	13536		
trnV	L	13537	13607		
12S rRNA	L	13608	14371		
trnI	H	14372	14435		
