# Supplementary material for: Unusual Genetic Diversity Within Thereuopoda clunifera (Wood, 1862) (Chilopoda: Scutigeromorpha) Revealed by Phylogeny and Divergence Times Using Mitochondrial Genomes
Source: Insects. 2025 May 2;16(5):486. doi: 10.3390/insects16050486 (PMC12112239; doi:10.3390/insects16050486)
Supplement: Supplementary file 1 [file insects-16-00486-s001.zip › Table S4.pdf]

Table S4. Base composition of 13 PCGs in seven mitogenomes.

Species	Region	whole genome	PCGs		tRNA		rRNA
	Strand	Heavy	Heavy	Light	Heavy	Light	Light
Thereuopoda clunifera GDSW04	length(bp)	14,899	6804	4287	821	566	1950
	AT%	69.3	66.3	70.6	73.8	69.6	71.9
	AT-skew	0.037	-0.082	-0.235	0.007	-0.071	-0.043
	GC-skew	-0.33	-0.311	0.392	-0.005	0.407	0.389
Thereuopoda clunifera ZJYY08	length(bp)	14,904	6810	4287	821	566	1953
	AT%	69.5	66.7	70.7	73.8	70.1	72.4
	AT-skew	0.03	-0.092	-0.23	0.01	-0.058	-0.036
	GC-skew	-0.315	-0.291	0.381	-0.014	0.396	0.383
Thereuopoda clunifera GXJX13	length(bp)	14,903	6798	4287	821	566	1952
	AT%	69.6	66.9	70.8	74	70.2	72.1
	AT-skew	0.035	-0.087	-0.235	0.008	-0.063	-0.046
	GC-skew	-0.323	-0.294	0.402	-0.019	0.396	0.385
Thereuopoda clunifera HBSZ18	length(bp)	14,897	6804	4287	820	568	1948
	AT%	69.8	66.9	71.7	74.2	70.5	71.7
	AT-skew	0.016	-0.115	-0.216	0.008	-0.075	-0.021
	GC-skew	-0.311	-0.281	0.393	-0.033	0.405	0.368
Thereuopoda clunifera GXGG22	length(bp)	14,898	6804	4287	822	564	1949
	AT%	68.1	64.9	69.5	74.5	69.5	71.2
	AT-skew	0.043	-0.097	-0.256	0.02	-0.061	-0.051
	GC-skew	-0.319	-0.296	0.382	-0.029	0.407	0.382
Thereuopoda clunifera HNCM23	length(bp)	14,900	6804	4287	822	564	1951
	AT%	69	66.1	70.2	73.9	70.4	71.5
	AT-skew	0.035	-0.098	-0.247	0.015	-0.053	-0.034

	GC-skew	-0.299	-0.281	0.361	-0.014	0.401	0.345
Thereuopoda clunifera HNWG24	length(bp)	14,898	6804	4287	820	568	1948
	AT%	69.8	66.7	72	74.1	70.1	72
	AT-skew	0.014	-0.116	-0.214	0.01	-0.07	-0.011
	GC-skew	-0.313	-0.285	0.397	-0.038	0.4	0.364
