# Supplementary material for: Unusual Genetic Diversity Within Thereuopoda clunifera (Wood, 1862) (Chilopoda: Scutigeromorpha) Revealed by Phylogeny and Divergence Times Using Mitochondrial Genomes
Source: Insects. 2025 May 2;16(5):486. doi: 10.3390/insects16050486 (PMC12112239; doi:10.3390/insects16050486)
Supplement: Supplementary file 1 [file insects-16-00486-s001.zip › Table S5.pdf]

Table S5. Results of species delimitation using bPTP based on the thirteen PCGs

Species	Specimens number	Support
1	*T.clunifera* GDSW04, *T.clunifera* GXJX13, *T.clunifera* OL436141, *T.clunifera* ZJYY08	0.924
2	*T.clunifera* HBSZ18, *T.clunifera* HNWG24	0.877
3	*T.clunifera* GXGG22	0.895
4	*T.clunifera* HNCM23	0.895
