# Supplementary figures and images for: Unusual Genetic Diversity Within Thereuopoda clunifera (Wood, 1862) (Chilopoda: Scutigeromorpha) Revealed by Phylogeny and Divergence Times Using Mitochondrial Genomes
Source: Insects. 2025 May 2;16(5):486. doi: 10.3390/insects16050486 (PMC12112239; doi:10.3390/insects16050486)

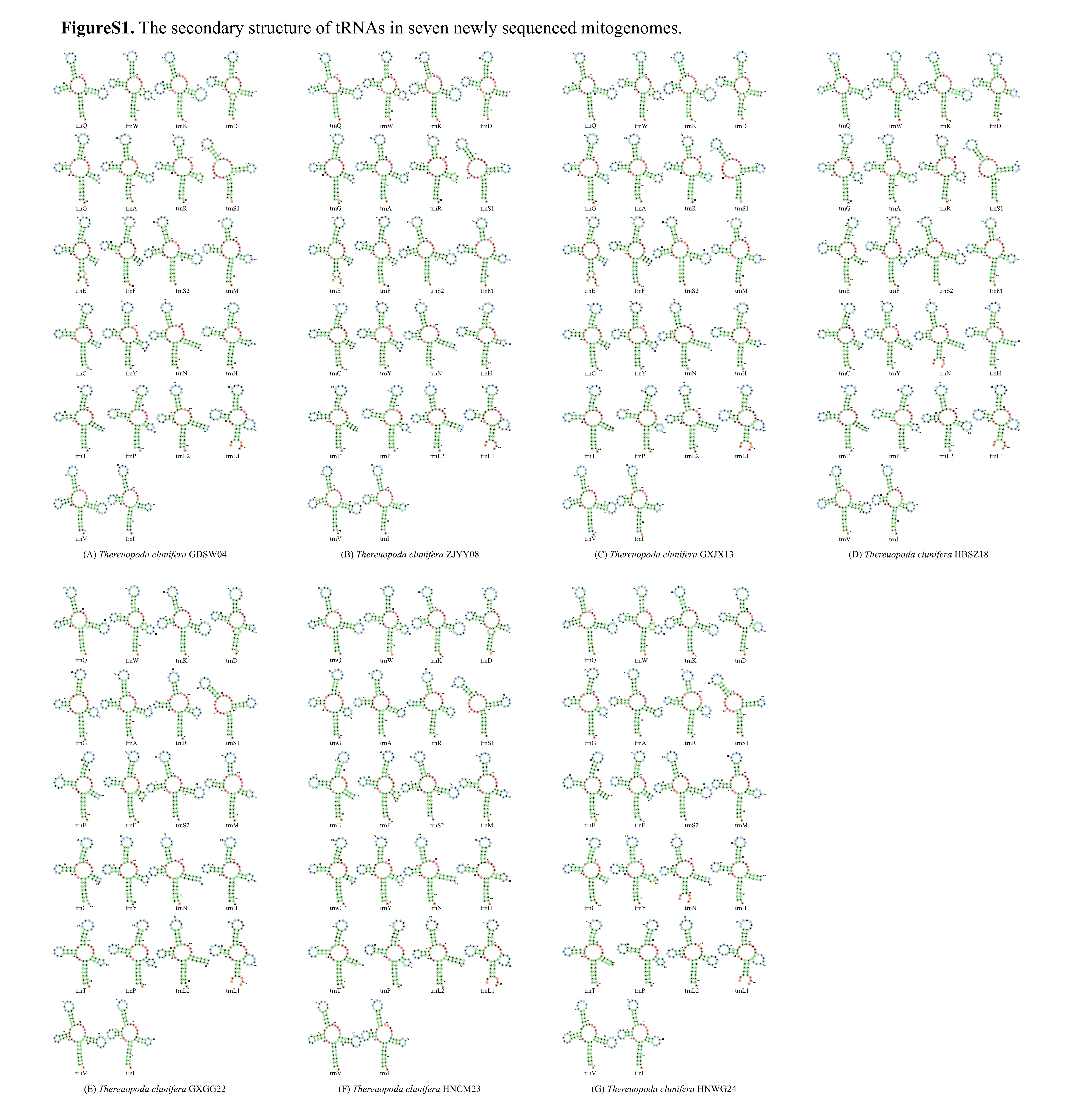

Supplement: Supplementary file 1 [file insects-16-00486-s001.zip › Figure S1.png]

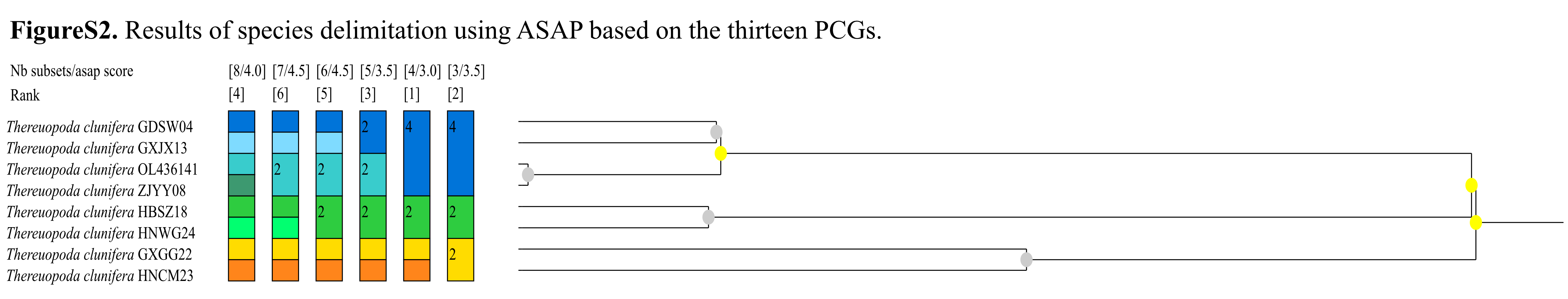

Supplement: Supplementary file 1 [file insects-16-00486-s001.zip › Figure S2.png]
